# Antibiotic prescribing in paediatric inpatients in Ghana: a multi-centre point prevalence survey

**DOI:** 10.1186/s12887-018-1367-5

**Published:** 2018-12-20

**Authors:** Appiah-Korang Labi, Noah Obeng-Nkrumah, Gifty Sunkwa-Mills, Antoinette Bediako-Bowan, Christiana Akufo, Stephanie Bjerrum, Enid Owusu, Christabel Enweronu-Laryea, Japheth Awuletey Opintan, Jorgen Anders Lindholm Kurtzhals, Mercy Jemima Newman

**Affiliations:** 1 Department of Immunology and Microbiology, University of Copenhagen, Copenhagen, Denmark; 2 Department of Microbiology, Korle-Bu Teaching Hospital, P.O. Box 77, Accra, Ghana; 3 Department of Medical Laboratory Sciences, School of Biomedical and Allied Health Sciences College of Health Sciences, University of Ghana, P.O. Box KB 143, Accra, Ghana; 4 Department of Public Health, Global Health Section, University of Copenhagen, Copenhagen, Denmark; 5 Department of Medical Microbiology, School of Biomedical and Allied Health Sciences, College of Health Sciences, University of Ghana, P.O. Box 143, Accra, Korle-Bu, Ghana; 6 Department of Animal Science, University of Copenhagen, Copenhagen, Denmark; 7 Department of Surgery, School of Medicine and Dentistry, College of Health Sciences, P. O. Box 4326, Accra, Ghana; 8 Department of Surgery, Korle-Bu Teaching Hospital, P.O. Box 77, Accra, Ghana; 9 Institutional Care Division, Ghana Health Service, PMB Ministries-Accra, Accra, Ghana; 10 Department of Child Health, School of Medicine and Dentistry, College of Health Sciences, University of Ghana, P.O.Box 4326, Accra, Ghana; 11 Centre for Medical Parasitology, Department of Clinical Microbiology, Copenhagen University Hospital (Rigshospitalet), Copenhagen, Denmark

**Keywords:** Antibiotic use, Children, Paediatric, Point prevalence, Ghana

## Abstract

**Background:**

Excessive and inappropriate use of antibiotics in hospitalised patients contributes to the development and spread of antibiotic resistance. Implementing a stewardship programme to curb the problem requires information on antibiotic use. This study describes a multicentre point prevalence of antibiotic use among paediatric inpatients in Ghana.

**Methods:**

Data were extracted from a multicentre point prevalence survey of hospital acquired infections in Ghana. Data were collected between September 2016 and December 2016 from ten hospitals through inpatient folder and chart reviews using European Centre for Disease Control (ECDC) adapted data collection instrument. From each site, data were collected within a 12-h period (8 am to 8 pm) by a primary team of research investigators and a select group of health professionals from each participating hospital.

**Results:**

Among 716 paediatric inpatients, 506 (70.6%; 95% confidence interval (CI): 67.2 to 74.0%) were on antibiotics. A significant proportion of antibiotics (82.9%) was prescribed for infants compared to neonates (63.9%) and adolescents (60.0%). The majority of patients (*n* = 251, 49.6%) were prescribed two antibiotics at the time of the survey. The top five classes of antibiotics prescribed were third generation cephalosporins (*n* = 154, 18.5%) aminoglycosides (*n* = 149, 17.9%), second generation cephalosporins (*n* = 103,12.4%), beta lactam resistant penicillins (*n* = 83, 10.0%) and nitroimidazoles (*n* = 82, 9.9%). The majority of antibiotics (*n* = 508, 61.0%) were prescribed for community acquired infections. The top three agents for managing community acquired infections were ceftriaxone (*n* = 97, 19.1%), gentamicin (*n* = 85, 16.7%) and cefuroxime (*n* = 73, 14.4%).

**Conclusion:**

This study points to high use of antibiotics among paediatric inpatients in Ghana. Cephalosporin use may offer an important target for reduction through antibiotic stewardship programmes.

## Background

Antibiotic resistance is a public health threat of global proportions [1]. It is associated with increased costs of health services, prolonged hospital stays and poor clinical outcomes [2, 3]. There are increasing reports of antibiotic resistance in hospital-acquired as well as community-acquired infections in low resource countries such as Ghana, with a significant number of cases reported among children [4–8]. This situation, if not urgently addressed, has the potential to jeopardize gains made in the management of infectious diseases due to shortfalls in healthcare financing, poor diagnostic capabilities and non-availability of newer therapeutic agents for the management of drug resistant bacteria [9].

Antibiotic resistance, though a natural phenomenon, has been accentuated by inappropriate use of antibiotics which selects for and contributes to the spread of resistant bacteria [10–13]. Strategies that have been proposed to counter antibiotic resistance include improved infection prevention and control programmes; surveillance of antimicrobial resistance; surveillance of antibiotic use; and implementation of antimicrobial stewardship programmes [1, 14–17].

To develop antimicrobial stewardship programmes, there is a need to acquire accurate baseline data on antibiotic prescription practice [18]. As an alternative to long term prospective surveillance that is usually not available in low resource countries, point prevalence surveys of antibiotic use have been found useful in generating such data [19–21]. This study describes antibiotic prescribing patterns among hospitalised children and adolescents in Ghana using data from a multicentre point prevalence survey conducted between September and December 2016 [22]. We describe antibiotic use across paediatric age groups and hospital levels, indications for antibiotic use, and targets for improved antibiotic use within the population.

## Methods

### Study design and population

The report presented here on antibiotic use in patients ≤18 years is a sub-analysis to a multicentre point prevalence survey of hospital acquired infections. The study design was a retrospective patient record review using data collection instrument. The study was conducted among in-patients at ten hospitals in Ghana between September and December 2016 [22]. Details of the methodology have been described elsewhere [22, 23]. Briefly, representative samples of acute care hospitals were selected, using systematic random sampling, according to size and hospital type (teaching/tertiary, regional/secondary, and district/primary hospitals). Each of the ten regions in Ghana was represented by one hospital. There are 3 teaching hospitals, 10 regional hospitals, and about 162 district hospitals in Ghana [24]. Together these hospitals register a bed occupancy rate of 60.4% (*n* = 8195) in 2017 for a total bed capacity of 12,806.

### Data collection

Data were collected from patient folders and treatment charts using standardised forms adapted from the European Centre for Disease Control (ECDC) [23]. Data recorded included type of antibiotic administered, route of administration, indication for use, number of doses per day, anatomic site targeted and duration of administration. Medical records of all patients on admission on or before 8 am on the day of the study were reviewed. Data collection was conducted within a 12-h period, i.e. 8 am to 8 pm. For hospitals with large bed capacity (> 1000), individual wards were investigated within a 12-h period and the total time frame for data collection at all wards of the hospital did not exceed one week. Data collection from each hospital was performed by two teams: a primary team of research investigators and a selected group of health professionals from the participating hospital.

### Definitions

Data on antibiotic use was recorded when a patient was actively on antibiotics at the time of the survey. As per ECDC guidelines, indication for antibiotic use was assigned to either community acquired infection (infection with symptoms occurring within 48 h of hospital admission) or hospital acquired infection (an active infection with signs and symptoms occurring after 48 h of admission or when the patient presented with an infection but had been readmitted less than two days after a previous discharge from a hospital). Unknown indications included antibiotic treatments with missing information on origin of active infection. Diagnosis of infection was categorised according to anatomic systems [23, 25]: eye; ear, nose, throat, larynx and mouth (ENT); respiratory tract (including pneumonia and acute bronchitis); cardiovascular system (CVS) including endocarditis and vascular grafts; gastrointestinal tract (GIT); intra-abdominal organs including those of the hepatobiliary system; skin, soft tissue, bone and joint (SSTBJ); central nervous system (CNS); genitourinary and gynaecological (GUOB); and those with undefined infection sites.

### Data handling and statistical analysis

Data was analysed using Access (Microsoft Office 2016) and Statistical Package for Social Sciences (SPSS, Version 21.0). Antibiotics were classified according to the Anatomical Therapeutic Chemical Classification System (ATC) [26]. We compared prevalence of antibiotic use across different hospital levels and across different age groups using the chi-square tests for multiple proportions with Marachuilo post hoc analysis. Two –tailed *p*-values < 0.05 were considered statistically significant. We categorized our study as neonates if ≤28 days; and infants if > 28 days to ≤2 years. In the Ghanaian healthcare system, patients aged 0 up to 13 years are attended to in paediatric departments whilst those > 13 years are seen at adult departments. We thus assigned the term children if aged > 2 years to ≤13 years, and adolescents if aged > 13 years to ≤18 years.

## Results

Data was collected from 2 tertiary hospitals, 3 secondary hospitals, and 5 primary hospitals (Table 1). The included hospitals had a total 4208 acute care beds, representing 32.9% of the total bed capacity for government hospitals in Ghana, and an overall bed occupancy of 59.1%. The median bed-size in a hospital was 304, ranging from 139 to 1533.

**Table 1:** Prevalence of antibiotic use by age and across type of hospital

Patient characteristics	Number of patients on		Prevalence of antibiotic use	
	Admission (%)	Antibiotics	Percent	95% CI
Age group^a^				
≤ 28 days	208 (29.1)	133	63.9	57.0–70.1
> 28 days to ≤2 years	152 (21.2)	126	82.9	75.8–88.3
> 2 years to ≤13 years	272 (40.0)	197	72.4	66.6–77.6
> 13 years to ≤18 years	84 (11.7)	50	60.0	48.2–69.9
Hospital type				
Tertiary	340 (47.5)	223	65.6	60.4–70.6
Regional	175 (24.4)	146	83.4	77.2–88.2
District	201 (28.1)	137	68.2	61.4–74.2
Total	716 (100.0)	506	70.6	67.2–74.0

^a^≤28 days, neonates; > 28 days to ≤2 years, infants; > 2 years to ≤13 years, children; > 13 years to ≤18 years, adolescents; %, percentage, *CI* confidence interval

### Hospital and patient characteristics

A total of 849 inpatients under the age of 18 years were included. Among these, 133 neonates were healthy and only in hospital because their mothers had not been discharged home. None of these received antibiotics and they were excluded from further analyses, leaving a total of 716 patients included for analysis. Baseline demographic data are shown in Table 1. Of the patients, 201 (28.1%) were from district hospitals, 175 (24.4%) from regional hospitals and 340 (47.5%) from tertiary hospitals. The average duration of stay of the patients prior to the survey was 8 days, this varied from 5 days in the regional hospitals to 6 days in district hospitals and 9 days in tertiary hospitals.

### Use of antibiotics

**Fig. 1:**
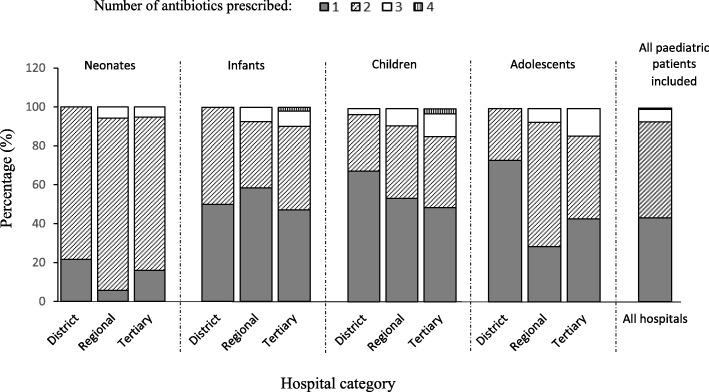
Number of antibiotics prescription per patients’age category across hospital type

Out of 716 patients on admission, 506 [70.6%, confidence interval (CI): 67.2 to 74.0] received at least one antibiotic at the time of the survey. There was a significant difference between the proportions of patients receiving antibiotics in regional hospitals (83.4%), tertiary (65.6%) and district hospitals (68.2%, *p* = 0.0001). The proportion of antibiotic use among infants (82.9%) was significantly higher compared to antibiotic use in neonates (63.9%) and adolescents (60.0%) but similar to the proportion of use among children (72.4%, p = 0.0001, Table 1). Of patients on antibiotics, the majority (*n* = 251, 49.6%) were prescribed two antibiotics whereas fewer received one antibiotic (*n* = 219, 43.3%), three antibiotics (*n* = 33, 6.5%) or four antibiotics (n = 3, 0.6%). This trend was observed among the neonatal, infant and adolescent populations, but not for children who were more likely to receive one antibiotic (Fig. 1). A total of 831 individual antibiotics were prescribed; 694 (83.5%) were administered via the intravenous route and 137 (16.5%) via the oral route, which was similar across district, regional and tertiary hospitals. Average duration of antibiotic administration at the time of the survey was 4 days, ranging between 1 and 13 days. Average duration of antibiotic administration was 3 days for district hospitals, 4 days for regional hospitals and 6 days for tertiary hospitals.

### Antibiotic utilisation at different types of healthcare facilities

**Fig. 2:**
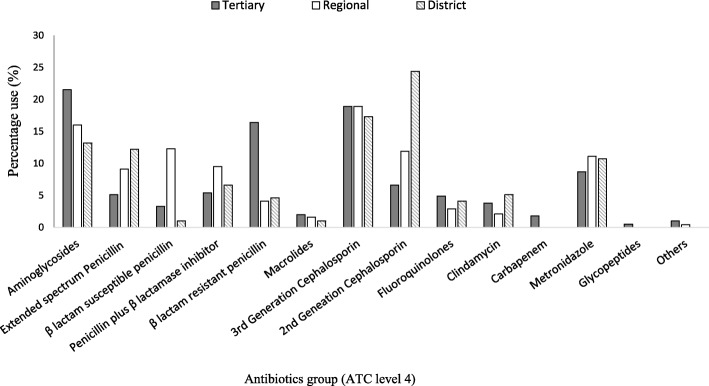
Antibiotic use by ATC level 4 across hospital category. Others include nitrofurantoin and co-trimoxazole

Figure 2 shows antibiotic use by class (ATC Level 4) across the different hospital types. The top five classes of antibiotics used (*N* = 831) were third generation cephalosporins (*n* = 154, 18.5%), aminoglycosides (*n* = 149, 17.9%), second generation cephalosporins (*n* = 103, 12.4%), beta lactam resistant penicillins (*n* = 83, 10.0%) and nitroimidazoles (*n* = 82, 9.9%). The proportion of third generation cephalosporin used was similar at the three hospital levels, i.e. tertiary (*n* = 74, 18.9%), regional (*n* = 46, 18.9%), district (*n* = 34, 17.3%, *p* = 0.9). Similar proportions of aminoglycosides were used in tertiary (*n* = 84, 21.5%) and regional (*n* = 39, 16%) hospitals, whereas significantly lower proportion were used in district hospitals (*n* = 26, 13.2%) compared to tertiary hospitals (*p* = 0.03). A significantly higher proportion of second generation cephalosporins were used in district hospitals (*n* = 48, 24.4%) compared to tertiary (n = 26, 6.6%) and regional hospitals (*n* = 29, 11.9%, *p* = 0.001). Carbapenems were only used in tertiary hospitals (*n* = 7, 0.8%). Of third generation cephalosporins prescribed, ceftriaxone was the commonest (*n* = 124, 80.5%) compared to ceftazidime (n = 1, 0.6%) and cefpodoxime (n = 1, 0.6%). Among the aminoglycosides, gentamicin (*n* = 114, 76.5%) and amikacin (*n* = 35, 23.5%) were the only prescribed agents, with amikacin being prescribed only in tertiary hospitals. Cefuroxime (*n* = 103) was the only prescribed second generation cephalosporin in all healthcare facilities. Cloxacillin (*n* = 83) and metronidazole (*n* = 82) represent the only agents used across all healthcare facilities in the beta lactam resistant penicillin and nitroimidazole classes respectively. Among carbapenems, meropenem was the only agent prescribed (n = 7) (Fig. 2).

### Antibiotic utilisation among different age groups

**Fig. 3:**
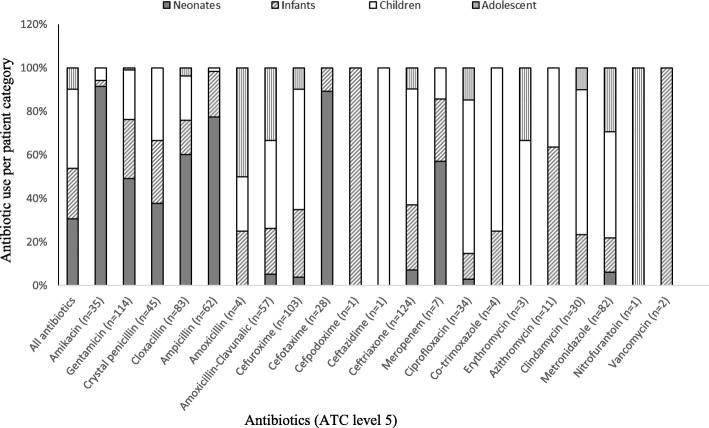
Proportion of antibiotic use (ATC level 5) across patients’ age category

The five most common antibiotics used among neonates were gentamicin (*n* = 56, 22%); cloxacillin (*n* = 50, 19.9%); ampicillin (n = 48, 18.9%); amikacin (*n* = 32, 12.6%); and cefotaxime (*n* = 25, 9.8%) (Fig. 3). Among infants, ceftriaxone (*n* = 37, 19.2%), cefuroxime (n = 32, 16.6%), gentamicin (*n* = 31, 16.1%), ampicillin (*n* = 13, 6.7%), crystal penicillin (n = 13, 6.7%) and metronidazole (n = 13, 6.7%) were the top six antibiotics used. Among children, the five most common antibiotics used were ceftriaxone (*n* = 66 (21.8%), cefuroxime (*n* = 57, 18.8%), gentamicin (*n* = 26, 8.5%), ciprofloxacin (*n* = 24, 7.9%) and amoxicillin-clavunalic acid (*n* = 23, 7.6%).

### Indication for antibiotic use

Out of the 831 antibiotics prescribed, 508 (61%) were prescribed for community-acquired infections, 86 (10.3%) for hospital-acquired infections, 197 (23.7%) for prophylaxis and 40 (4.8%) for unknown reason (Table 2). The top three agents for managing community-acquired infections were ceftriaxone (*n* = 97, 19.1%), gentamicin (*n* = 85, 16.7%) and cefuroxime (*n* = 73, 14.4%). The common agents used for surgical prophylaxis were metronidazole (*n* = 20; 28.2%), amoxicillin-clavulanic acid (*n* = 15, 21.1%) and cefuroxime (n = 13, 18.3%).

**Table 2:** Indications for prescribing antimicrobial drugs (ATC^a^ level 5) and the five most common antibiotics used

Indication for antibiotic use	Top 5 antibiotic use among paediatric inpatients				
	1^st^	2^nd^	3^rd^	4^th^	5^th^
All indications (*n* = 831)	Ceftriaxone (*n* = 124, 14.9%)	Gentamicin(n = 114, 13.7%)	Cefuroxime (*n* = 103, 12.4%)	Cloxacillin (n = 83, 9.9%)	Metronidazole (n = 82, 9.8%)
Community acquired infections (n = 508)	Ceftriaxone (n = 97, 19.1%)	Gentamicin (n = 85,16.7%)	Cefuroxime (n = 73, 14.4%)	Cloxacillin (*n* = 48, 9.4%)	Metronidazole (*n* = 47, 9.3%)
Hosptal acquired infections (*n* = 86)	*Cloxacillin* (*n* = 12, 14.0%)	Gentamicin (n = 12, 14.0%)	Cefuroxime (n = 8, 9.3%)	Ciprofloxacin(n = 7, 8.1%)	Amikacin (n = 6, 7.0%)
					Ampicillin
					Ceftriaxone
					Meropenem
Medical prophylaxis (*n* = 126)	Ampicillin (*n* = 17, 13.5%)	Gentamicin (*n* = 1, 13.3%)	Cefotaxime (*n* = 16, 12.7%)	Amikacin (n = 14, 11.1%)	Crystal penicillin (n = 13, 10.3%)
	Cloxacillin				
Surgical prophylaxis (*n* = 71)	Metronidazole (n = 20, 28.2%)	Amoxicillin-clavulanic acid (n = 15, 21.1%)	Cefuroxime (n = 13, 18.3%)	Ceftriaxone (n = 7,9.9%)	Ampicillin (n = 4, 5.6%)
					Ciprofloxacin
Unknown (*n* = 40)	Ceftriaxone (n = 8, 20.0%)	Amox-clavulanic acid (n = 4, 10.0%)	Metronidazole (n = 3, 7.5%)	Amikacin (n = 2, 5.0%)	Erythromycin (n = 1, 2.5%)
			Ciprofloxacin	Cefuroxime	Azithromycin
			Clindamycin	Crystal penicillin	Co-trimoxazole
			Ampicillin		Amoxicillin
			Cloxacillin		
			Gentamicin		

^a^*ATC* Anatomical Therapeutic Chemical Classification System

Based on the anatomic site, antibiotics were mainly prescribed for undefined reasons (*n* = 328, 39.5%), followed by the respiratory system (*n* = 196, 23.6%) and skin, soft tissue, bone and joint infections (*n* = 131, 15.8%) (Table 3). Infection without a defined focus formed the majority of the undefined reasons for antibiotic use in neonates. The top three antibiotics used in managing infections with undefined focus were gentamicin (*n* = 59, 18.0%), ceftriaxone (n = 57, 17.4%) and ampicillin (*n* = 45, 13.7%). For respiratory tract infections, gentamicin (*n* = 35, 17.9%), cefuroxime (*n* = 34, 17.3%) and ceftriaxone (n = 2, 14.8%) were the three most commonly used antibiotics. The top three antibiotics prescribed for skin, soft tissue, bone and joint purposes were metronidazole (*n* = 24, 18.3%), cloxacillin (*n* = 23, 17.6%) and clindamycin (n = 20, 15.3%).

**Table 3:** Anatomic sites of infection and the five most common antimicrobial drugs (ATC level 5) prescribed

Anatomic sites^a^	Top 5 antibiotic use (ATC^a^ level 5) among paediatric patients				
	1^st^	2^nd^	3^rd^	4^th^	5^th^
Resp (n = 196)	Gentamicin (n = 35, 17.9%)	Cefuroxime (n = 34, 17.3%)	Ceftriaxone (n = 29, 14.8%)	Amox-clav (*n* = 18, 9.2%)	Cloxacillin (n = 12, 6.1%)
				C penicillin	
SSTBJ (n = 131)	Metronidazole (n = 24,18.3%)	Cloxacillin (n = 23, 17.6%)	Clindamycin (n = 20, 15.3%)	Cefuroxime (n = 19, 14.5%)	Amox-clav (n = 17, 13.0%)
GIT (*n* = 88)	Metronidazole (n = 29, 32.9%)	Cefuroxime (n = 17,19.3%)	Ciprofloxacin (n = 13,14.8%)	Ceftriaxone (n = 11,12.5%)	Gentamicin (n = 7, 8.0%)
GUOB (n = 33)	Metronidazole (n = 8,24.2%)	Amox-clav (n = 7, 21.2%)	Cefuroxime (n = 5, 15.2%)	Ceftriaxone (n = 4, 12.1%)	Ciprofloxacin (n = 3, 9.1%)
CNS (n = 26)	Amox-clav^a^ (n = 2, 7.7%)	Ceftriaxone (n = 11, 3.4%)	Cloxacillin (n = 5, 1.5%)	Metronidazole (n = 3,0.9%)	Gentamicin (n = 1, 0.3%)
	Cefuroxime				Cefotaxime
					Meropenem
UTI (n = 14)	Cefuroxime (n = 5, 35.7%)	Ciprofloxacin (n = 3, 21.4%)	Other drugs^b^ (*n* = 6, 43.8%)	–	–
Other sites (n = 15)	Ceftriaxone (n = 4, 26.6%)	Amox-clav (n = 2, 13.1%)	Other drugs^c^ (n = 4, 26.6%)	–	–
	C penicillin^a^				
UND (n = 328)	Gentamicin (n = 59,18.0%)	Ceftriaxone (*n* = 57, 17.4%)	Ampicillin (n = 45,13.7%)	Cloxacillin (*n* = 42,12.8%)	Amikacin (n = 25,7.6%)

^a^*Resp* respiratory tract, *GIT* Gastrointestinal tract, *UTI* Urinary tract infection, *CNS* Central nervous system, *SSTBJ* skin, soft tissue, bones and joints, *GUOB* Genitourinary and obstetrics; Other sites comprise cardiovascular system and Ear, nose and throat; *UND* Undefined, *Amox-clav* Amoxicillin-clavulanic acid, *C pencillin* Crystal penicillin, *ATC* Anatomical Therapeutic Chemical Classification System

^b^other drugs include crystal penicillin (n = 1), ceftriaxone (n = 1), gentamicin (n = 1), meropenem (n = 1), nitrofurantoin (n = 1), erythromycin (n = 1)

^c^others include crystal penicillin (n = 1), ceftriaxone (n = 1), gentamicin (n = 1), meropenem (n = 1), nitrofurantoin (n = 1), erythromycin (n = 1)

## Discussion

To the best of our knowledge this study presents one of the few multicentre studies on antibiotic use among hospitalised paediatric patients from Africa and the first from Ghana. It provides current patterns of antibiotic prescribing practices in this population in Ghana and can serve as a baseline for future comparison. We observed that 71% of the hospitalised paediatric and adolescent patients were prescribed antibiotics with a high percentage of use among infants and neonates. This figure is comparable to a 69.4% rate of antibiotic use observed among children in a recent single site study from Korle-bu Teaching Hospital in Ghana [27]. The rate is comparable to findings from the United States of America [28], but higher than 36.7% recorded in a recent worldwide survey of antibiotic use in children [29].

The lowest antibiotic use was reported for tertiary hospitals. This may be attributed to the fact that doctors in tertiary hospitals have more opportunities for rational antibiotic use: through participation in continuing education, consultations with senior physicians, and research [1, 14–17, 30]. Although we accept the possibility that doctors’ practices on antibiotic use in tertiary hospitals may still be insufficient [27], it is notable that doctors coming from primary healthcare institutions have poorer knowledge, attitudes and practices on antibiotic use [31, 32]. Analysis of data from the Chinese Nosocomial Infection Control Surveillance Networks reveals that, compared with large secondary and tertiary hospitals, excessive and improper use of antibiotics is more severe in primary health care facilities [33, 34]. In some European Countries, published data suggest higher antibiotic use in tertiary health facilities [25]. The heterogeneity in the level antibiotic use across hospital types is highlighted in the recent study that reviewed published works and online surveillance reports on antibiotic consumption in acute care hospitals between the years 1997 and 2013 [35]. In their pooled estimate for antibiotic consumption, there was a non-significant trend for higher antibiotic consumption in tertiary compared to non-tertiary hospitals.

In our study, we observed a very high rate of parenteral antibiotic use, especially in tertiary hospitals. It has been suggested that high rates of parenteral antibiotic use persists among children because of limited options for oral broad spectrum antibiotics with appropriate formulations and the challenges of oral administration of medications in young children [36]. Similar high rates of parenteral antibiotic use have been observed in other settings [18, 27]. Parenteral antibiotic use is a known quality indicator of antibiotic prescribing practices [21, 37]. Low rates of intravenous antibiotic use are preferred since they are associated with lower levels of thrombophlebitis, cannula related infections, lower cost of care and early discharge [37, 38]. There was a high usage of third and second generation cephalosporins as well as aminoglycosides among patients in all types of healthcare facilities in the country. This may be due to the abundance of cheap generic options on the market, as well as their availability on the national health insurance scheme at all levels of the health care system [39]. Globally, cephalosporins are used empirically in patient care due to their low toxicity, they are however known to contribute to the development and spread of multi-drug resistant infections [40]. Extensive use of third generation cephalosporins among primary and secondary level facilities may be contributing to the rise and spread of extended spectrum beta lactamase-producing bacteria in Ghanaian hospitals [4, 41]. This phenomenon may limit the usefulness of these agents at the tertiary level in managing referred cases. Low prescribing rates of antibiotics such as carbapenems and vancomycin were observed from this study. These agents were only used in tertiary healthcare facilities and mainly in one healthcare facility, the Korle-Bu Teaching Hospital. Low prescription rates of these agents could be accounted for by their unavailability on the national health insurance scheme and the high cost associated with out of pocket purchase [39]. This practice has the potential to slow down the development and spread of carbapenemase-producing and vancomycin-resistant bacteria among paediatric patients.

The highest use of gentamicin, amikacin, ampicillin and cloxacillin was in the neonatal age group. These agents are recommended by the standard treatment guidelines of Ghana and by other international guidelines for neonatal infections [42, 43]. Our data suggest a strong adherence to the recommended treatment protocols among the neonatal age group. Similar findings have been reported in recent studies on antibiotic use among children [29, 44, 45]. Amikacin use in this study was accounted for by one tertiary healthcare facility i.e. Korle-Bu Teaching Hospital. In this facility, amikacin in combination with cloxacillin forms the standard treatment protocol for infections in the neonatal intensive care unit. This protocol has been in place for the past 6 years and was instituted after a review of the unit’s antibiogram showed increasing resistance to ampicillin/gentamicin combination which was the previously recommended standard treatment [46]. In contrast, there was a sharp difference in the classes of antibiotics used among the other age groups, they were mainly treated with broad spectrum agents such as third and second generation cephalosporins. Antibiotics were commonly prescribed for infections of undefined origin, in particular among neonates. This may reflect challenges with diagnosing infections in neonates with a resultant high rates of empirical antibiotic use.

The use of broad spectrum antibiotics among children is a quality indicator for antibiotic prescribing as it can lead to the development and spread of antibiotic resistance [29]. Use of such broad spectrum antibiotics in children may reflect the lack of adherence to suggested standard treatment guidelines. The prescribing pattern may also be affected by the relatively low utilisation of microbiology culture services observed in an earlier study [22]. The limited availability and usage of clinical microbiology services thus promotes empirical antibiotic prescribing against susceptibility guided therapy; it also does not allow for stepping treatment down from broader to narrower spectrum antibiotics. Several studies in Ghana have shown an increase in the incidence of infections caused by extended spectrum beta lactamase-producing bacteria. This may be a direct result of the high usage of cephalosporins [4, 6, 41, 47]. Metronidazole, amoxicillin clavulanic acid and cefuroxime were the most common antibiotics used for surgical prophylaxis. High use of metronidazole as a prophylactic agent in surgery practice has been documented in other studies in Ghana [27, 48]. Metronidazole used in combination with antibiotics with aerobic coverage has been found to prevent surgical site infections in abdominal surgeries [49, 50]. Although this study did not assess the appropriateness of antibiotic prescribing there is evidence that metronidazole amoxicillin-clavulanic acid combination is commonly used despite the fact that it represents double cover for anaerobic bacteria.

Reducing antibiotic consumption through stewardship programmes is a major global strategy being advanced to curb the development and spread of antibiotic resistance [14, 51–55]. In resource limited countries like Ghana, third generation cephalosporins represent a safe broad-spectrum agent useful for the management of very severe Gram negative and positive infections. Our study highlights third generation cephalosporins as a major antibiotic class for which antibiotic stewardship strategies are urgently needed to preserve their usefulness among hospitalised paediatric patients. Failure to preserve these agents may result in increased use of newer and more expensive agents like carbapenems and glycopeptides due to growing antibiotic resistance; a situation which may lead to a reversal of gains made in the management and control of infectious diseases. There is also the need for improved access to diagnostic clinical microbiology services which would contribute to improved diagnosis and management of conditions such as sepsis and lead to a reduction in antibiotic use.

This study has some potential limitations. Although the data presented here gives a good situation report of publicly funded health facilities, findings may not be directly extended to private healthcare facilities. We recorded the duration of antibiotic treatment, but the appropriateness and length of antibiotic use was not assessed at the individual level, nor was the indication for culture before antibiotic therapy. This could have provided significant information to guide antibiotic stewardships strategies and may be considered in future surveys. Another limitation is the study’s time window. Being a point prevalence study, information on antibiotic use was collected at a single point in time over the period between September and December 2016. This design would not capture the seasonality of infections and antibiotic prescriptions. Conducting this survey throughout multiple time points would have allowed for a more robust estimate of inpatient antibiotic prescribing in Ghana. However, we did not have the necessary capacity to repeat this multi-site point prevalence study more than once. Despite these limitations we believe the use of standardized international protocol [23] for our study gives us robust evidence of antibiotic use among hospitalized paediatric inpatients in Ghana and serves as a bench mark for future comparison.

## Conclusion

Our study shows high use of antibiotics among paediatric inpatients in Ghana. It also highlights key differences in antibiotic use among neonates and the other paediatric population; neonates are likely to be prescribed an aminoglycoside and a penicillin as opposed to the dominant use of cephalosporins among the other age groups. Studies of the appropriateness of antibiotic use are urgently needed to guide antibiotic stewardship strategies aiming at reducing the use of agents such as cephalosporins in order to conserve their lifespan.

## Abbreviations

CI: Confidence interval
ECDC: European Center for Disease Prevention and Control
HAI: Healthcare associated infection
IQR: Interquartile range
KBTH: Korle-Bu Teaching Hospital
PPS: Point prevalence study
WHO: World Health Organization
